# Through the Eyes of Patients: The Effect of Training General Practitioners and Nurses on Perceived Shared Decision-Making Support

**DOI:** 10.1177/0272989X231203693

**Published:** 2023-10-24

**Authors:** Danique W. Bos – van den Hoek, Ellen M. A. Smets, Rania Ali, Dorien Tange, Hanneke W. M. van Laarhoven, Inge Henselmans

**Affiliations:** Amsterdam UMC location University of Amsterdam, Department of Medical Psychology, Amsterdam, The Netherlands; Amsterdam Public Health Research Institute, Quality of Care Program, Amsterdam, The Netherlands; Cancer Center Amsterdam, Cancer Treatment and Quality of Life, Amsterdam, The Netherlands; Amsterdam UMC location University of Amsterdam, Department of Medical Psychology, Amsterdam, The Netherlands; Amsterdam Public Health Research Institute, Quality of Care Program, Amsterdam, The Netherlands; Cancer Center Amsterdam, Cancer Treatment and Quality of Life, Amsterdam, The Netherlands; Amsterdam UMC location University of Amsterdam, Department of Medical Psychology, Amsterdam, The Netherlands; Dutch Federation of Cancer Patient Organizations (NFK), Utrecht, The Netherlands; Cancer Center Amsterdam, Cancer Treatment and Quality of Life, Amsterdam, The Netherlands; Amsterdam UMC location University of Amsterdam, Department of Medical Oncology, Amsterdam, The Netherlands; Amsterdam UMC location University of Amsterdam, Department of Medical Psychology, Amsterdam, The Netherlands; Amsterdam Public Health Research Institute, Quality of Care Program, Amsterdam, The Netherlands; Cancer Center Amsterdam, Cancer Treatment and Quality of Life, Amsterdam, The Netherlands; Amsterdam UMC Location University of Amsterdam, Department of General Practice, Amsterdam, The Netherlands

**Keywords:** shared decision making, continuing education, palliative care, neoplasms, patients, cancer survivors, general practitioners, nurses, communication

## Abstract

**Purpose:**

To examine the effects of training general practitioners and nurses in shared decision-making (SDM) support as perceived by cancer patients and survivors.

**Design:**

An innovative, experimental design was adopted that included analogue patients (APs), that is, people who have or have had cancer and who imagine themselves in the position of the actor-patient presented in a video. Each AP assessed a video-recorded simulated consultation of a health care professional (HCP) conducted before or after an SDM support training program. The primary outcome was the APs’ perceived SDM support with 13 self-developed items reflecting the perceived patient benefit of SDM support as well as the perceived HCP support behavior. Secondary outcomes included an overall rating of SDM support, AP-reported extent of SDM (CollaboRATE), satisfaction with the communication (Patient Satisfaction Questionnaire), conversation appreciation and helpfulness, as well as decision-making satisfaction and confidence (visual analog scale, 0–100). In addition, patient and HCP characteristics associated with AP-perceived SDM support were examined.

**Results:**

APs (*n* = 131) did not significantly differentiate trained from untrained HCPs in their perceptions of SDM support nor in secondary outcomes. Agreement between APs’ perceptions was poor. The higher the perceived comparability of the consultation with APs’ previous personal experiences, the higher their rating of SDM support.

**Limitations:**

We used a nonvalidated primary outcome and an innovative study design that should be tested in future work.

**Conclusions:**

Despite the limitations of the study design, the training seemed to not affect cancer patients’ and survivors’ perceived SDM support.

**Implications:**

The clinical relevance of the training on SDM support needs to be established. The variation in APs’ assessments suggests patients differ in their perception of SDM support, stressing the importance of patient-tailored SDM support.

**Highlights:**

- Cancer patients and survivors did not significantly differentiate trained from untrained HCPs when evaluating SDM support, and agreement between their perceptions was poor.
- The clinical relevance of training GPs and nurses in SDM support needs to be established.
- Patient-tailored SDM support may be recommended, given the variation in APs’ assessments and their possible diverging perceptions of SDM support.
- This innovative study design (having patients watch and assess videos of simulated consultations made in the context of training evaluation) needs to be further developed.

Advanced cancer patients may need to make treatment decisions that depend on their personal evaluation of the benefits and harms of treatment options and hence require shared decision making (SDM). For example, they may need to choose between starting or forgoing systemic therapy, as treatment outcomes are uncertain and possibly limited while the burden of side effects may be high.^1,2^ SDM involves both the health care professional (HCP) and the patient, exchanging information about treatment options as well as patient values and preferences, to reach consensus about the preferred treatment.^3–6^ Patients generally make decisions about cancer treatment with medical specialists. However, general practitioners (GPs) and nurses may have a complementary, supporting role in SDM.^7–10^ A recent survey among patients and survivors showed that they appreciate the involvement of GPs and nurses after a cancer diagnosis.^11,12^

Attention for such interprofessional SDM and decision support by GPs and nurses has increased over the past years.^13–15^ Previous studies have identified 3 strategies HCPs may deploy to support SDM: 1) checking the quality of a decision (i.e., exploring whether patients are conscious of the existence of a decision, informed about the different options, and whether their values and preferences are incorporated into the treatment decision), 2) complementing SDM (i.e., contributing to the SDM process by increasing choice awareness, clarifying information, or supporting preference construction), and 3) enabling SDM (i.e., organizing activities to ensure that the SDM process will continue beyond HCPs’ direct involvement).^16,17^

Research shows that training can strengthen SDM support knowledge and skills of HCPs.^18–21^ We previously evaluated a training program in SDM support for GPs and nurses by having expert observers assess video-recorded simulated consultations conducted before and after the training. These observers established a medium-sized significant improvement in SDM support behavior after training.^21^ Former research, however, has shown that patients and trained observers evaluate the SDM behavior of clinicians differently.^22,23^ This stresses the necessity of exploring patient perspectives. Little is known about if and how patients experience support of GPs and nurses in decision making about cancer treatment after such training programs. While SDM support conversations might help patients make decisions,^24^ such attempts of HCPs might also go unnoticed or not facilitate or even hinder making a high-quality decision.^25^ Acquiring insights into whether a training program for GPs and nurses affects not only ratings of trained observers but also patients’ perceptions of SDM support would help understand whether the training program may indeed benefit patients in making treatment decisions and is therefore clinically relevant.

The aim of this study is to examine the effect of the training program for GPs and nurses on cancer patients’ and survivors’ perceived SDM support when assessing video-recorded simulated consultations. The primary outcome is SDM support as perceived by these so-called analogue patients (APs), which includes the perceived degree of SDM supportive behaviors of HCPs and the perceived benefit of this behavior for patients to feel more empowered to make a treatment decision. Secondary outcomes included self-reported SDM, satisfaction with the communication, conversation appreciation and helpfulness as well as decision-making satisfaction and confidence, and an overall rating of SDM support. In addition, the study aimed to examine which AP and HCP characteristics are associated with AP-perceived SDM support.

## Methods

### Design

**Figure 1:**
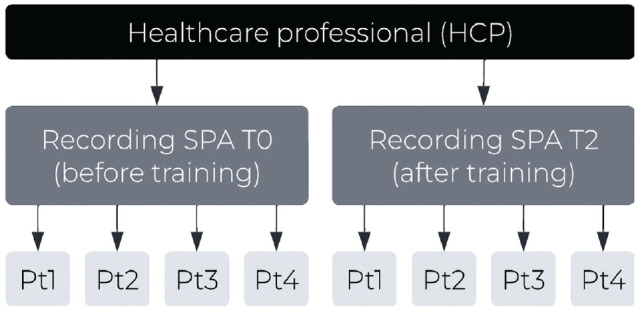
Study design.

We previously conducted standardized patient assessments (SPAs) before and after a training program on SDM support for GPs and nurses.^21^ The video-recorded SPAs were assessed by trained observers to evaluate the trainings’ effectiveness. In the current innovative, experimental study design, video recordings (*n* = 32) of SPAs of GPs (*n* = 8) and nurses (*n* = 8) conducted before (SPA T0) and after (SPA T2) the training were assessed by at least 4 cancer patients or survivors (*n* = 132); each participant watched 1 SPA. See Box 1 for more information on the SPAs and Figure 1 for the study design. The assessments took place between June 2021 and March 2022. The STROBE guidelines^26^ were followed where applicable in this report. The funding source had no role in the study.

**Box 1.** Standardized Patient Assessments (SPAs)

Health care professionals (HCPs) in the video-recorded standardized patient assessments (SPAs) were either general practitioners (GPs) or hospital nurses. GPs were GP educators who participate in continuing medical education meetings at GP training centers of Dutch academic hospitals. Nurses had an oncology specialization and cared for patients with cancer in a Dutch hospital. Seventy percent were clinical nurse specialists. In the SPAs, GPs met a patient with either metastatic gastric (case 1) or esophageal (case 2) cancer. The patient had had a conversation with the oncologist about starting palliative chemotherapy. Nurses met a patient with metastatic colorectal cancer (both cases) who just had a conversation with the oncologist regarding the results of the latest scan, which turned out to be poor, and the start of second-line palliative chemotherapy. Although the personal background of the patient cases differed, the medical situation was rather similar. See Appendix A for more details. Cases were randomly assigned to the SPA either before or after training. Before the start of the SPA, HCPs received a simulated specialist’s letter (GPs) or medical file (nurses) containing standard medical information. Three experienced professional male actors played all of the roles (actor A: 63 y; actor B: 57 y; actor C: 64 y). They were trained and instructed to act in a standard way, to be rather passive and not overly emotional. As a result of COVID-19 related restrictions, all SPAs took place online.

### Setting, Participants, and Recruitment

The study was powered to establish a medium effect (i.e., participants’ ability to discriminate between trained and untrained HCPs on SDM support) of the training program (Cohen’s *d* = 0.5) and required 128 participants (G*Power, α = 0.05, β = 0.80, *t* test). Participants were eligible if they were (previously) diagnosed with cancer and at least 50 y old, to increase engagement with the patient in the SPA. Cancer patients and survivors acted as APs (i.e., untrained viewers providing their perception of the interaction while taking on the patient role).^27^ APs can be used if real patient perceptions are not possible but one is interested in subjective patient perceptions,^27,28^ as APs’ and clinical patients’ perceptions of communication largely overlap.^28^ Participants were recruited through the patient panels of PanelCom and Kanker.nl, both of which target (ex)cancer patients. Snowballing was also applied by asking participants who had completed the study to invite eligible people in their network. Recruitment continued until at least 128 participants completed a questionnaire. Invitation e-mails were sent, and interested participants received participant information prior to receiving the online questionnaire invitation. The questionnaire required all participants to provide informed consent and sign a confidentiality agreement. Twenty gift vouchers were raffled among the participants. The Human Ethics Committee at the Amsterdam UMC location AMC provided ethical clearance for the study (W20_168 # 20.199).

### Study Procedures

A random selection of video-recorded SPAs (before and after training) from 8 GPs and 8 nurses was made, after verifying HCPs’ consent and checking the quality of the video-recorded SPAs. The video recordings were anonymized and uploaded in a safe digital environment. The APs’ online questionnaire contained a Web link to the recorded SPA that was randomly assigned to them. They first reported baseline characteristics and were subsequently instructed that they were going to watch a simulated conversation between an HCP and a patient (an actor). Personal and medical background information of the patient case was shared briefly as well as the context in which the conversation took place. APs were explicitly asked to not only observe but also sympathize with the actor-patient, that is, to put oneself in the shoes of the patient while watching the video recording and to pay particular attention to how the HCP and patient talked about the treatment decision. After watching the SPA, APs assessed primary and secondary outcomes in the questionnaire. Afterward, they could voluntarily express any comments about the study, questionnaire, or video recording in an open answer field. All but this last part of the questionnaire were mandatory to progress through the questionnaire.

### Measurements

#### Baseline characteristics

APs reported their age (in categories), sex, educational level, and medical background, that is, whether they have (had) cancer (distinguishing patients and survivors), whether this cancer is curable, year of (first) diagnosis, type of cancer, and type of treatment. In addition, APs’ preferred decisional role was measured with the Control Preference Scale (a 1-item measure with 5 different treatment decision-making roles^29^), which was adjusted for analyses to reflect an active, shared, or passive patient role.^30^ APs’ attitude toward striving for quantity or quality of life was assessed with the Quantity Quality Questionnaire, an 8-item questionnaire for assessing patient attitudes concerning tradeoffs between quality of life and quantity of life.^31^ Responses were given on a 5-point Likert scale (1 = *I strongly disagree* to 5 = *I strongly agree*). Two subscales were constructed by adding the respective item scores: striving for length of life and quality of life (4 items each). Lastly, 1 item assessed whether APs ever had such a conversation about the cancer treatment decision with their GP or nurse (yes/no), and, if yes, another item assessed APs’ perception of the comparability of the SPA with their own experience (1 = *not at all* to 10 = *very much*).

#### Fidelity and validity check

The digital environment in which APs watched the video-recorded SPAs provided analytic information on how many times the SPA was watched and for how long, in quartiles. This information was reviewed to gain insight in the fidelity of viewing SPAs but could not be linked to APs’ identification numbers. Moreover, interpretation should be cautious, as missing (parts of) viewings could also mean the AP did not turn on cookies or had Internet connection issues. Regarding the validity of the procedure, engagement with the video was measured, using the validated 9-item Dutch shortened Video Engagement Scale (VES-sf).^32,33^ This reflected “the extent to which participants are able to view, immerse and imagine themselves being in the video and potentially being emotionally touched by the video.”^32,33^ One item relating to whether participants paid attention was used as a screener, as this was considered a prerequisite to engage in the research and may bias results if too low.^32^ Items were rated on a 7-point Likert scale (1 = *completely disagree* to 7 = *completely agree*) and averaged, with higher scores reflecting greater engagement. In addition, 2 subscales were constructed: immersion and emotional impact, both of which were calculated by averaging the corresponding 4 items.^32^

#### Primary outcome

Because no patient-reported instrument on SDM support exists, 13 self-developed items were used to assess SDM support (Appendix B). Six items focused on the perceived *HCP behavior*, based on the 3 strategies of SDM support^16,17^: checking the quality of a decision (1 item), complementing SDM (4 items), and enabling SDM (1 item). An example was “In this conversation, the HCP helped to make it clear that there is a choice between several treatment options.” Seven items focused on the perceived *patient benefit* of SDM support (i.e., being more empowered to make a treatment decision after the consultation). These benefits referred to 3 aspects important for making high-quality treatment decisions^34^: being choice aware (2 items), informed (2 items), and able to make a decision aligned with patient values (3 items). For example, 1 item stated, “If you were the patient, after this conversation it would be clearer to you what you think of the pros and cons of the treatment(s).” All items were rated on a 6-point Likert scale (0 = *completely disagree* to 5 = *completely agree*). Two total scores were calculated: *SDM support – HCP behavior* and *SDM support – patient benefit*. These were calculated by averaging the items corresponding to each strategy or quality aspect, respectively, and subsequently averaging these 3 scores (range 0–5). The items were pilot tested with the first 4 participants, which resulted in some small refinements only.

#### Secondary outcomes

Next to self-developed items, the validated 3-item CollaboRATE^35^ was administered, which assesses *patient-reported SDM*. The items (e.g., “how much effort was made to include what matters to you most in choosing what to do next”) were rated on a 10-point Likert scale (0 = *made no effort at all* to 9 = *made every effort*), summed, and transformed to reflect a score on a range of 0 to 100. Second, *satisfaction with the communication in the consultation* was assessed with the 5-item Patient Satisfaction Questionnaire (PSQ).^36–38^ Items were slightly adapted to fit the AP setting (e.g., how well the HCP addressed the needs of the patient) and assessed on a visual analog scale (VAS; range 0–100). The total score of the PSQ was the average of the 5 items (range 0–100). Four additional study-specific items focused on the participants’ *decision-making satisfaction* (i.e., how satisfied they would be with the way the HCP spoke about the treatment decision), *conversation appreciation* (i.e., how much they would appreciate this conversation with the HCP), perceived *conversation helpfulness* (i.e., to what extent would the conversation with the HCP have helped in making a treatment decision), and *decision-making confidence* (i.e., how confident they are that a good decision has been or will be made). These items were also assessed on a VAS (range 0–100). Lastly, 1 global item assessed the overall rating of SDM support (i.e., the degree to which the HCP assisted the patient in making an appropriate decision) with a Likert scale response (1 = *not at all* to 10 = *to a very strong degree*).

### Statistical Analyses

First, linear mixed models (LMMs) were computed for exploring whether APs differentiated trained from untrained HCPs while accounting for the hierarchical structure of the data (cancer patients and survivors [level 1] within training condition [time, level 2] within HCPs [level 3]). There were no missing data. A random factor for the HCP level was used. If the variance between HCPs was too low, this random factor was removed, and AP assessments were treated as independent assessments that were not nested. Effect size was presented by Cohen’s *d* (*d* = 0.20 small, *d* = 0.50 medium, *d* = 0.80 large effects^39^). In addition, the intraclass correlations between APs’ assessments were calculated with 1-way random-effects models as each SPA was assessed by a different set of raters.^40^ Also, correlations between APs’ assessments and trained observers’ assessments from the previous study^21^ were calculated. Next, HCP discipline and its interaction with training status (HCP discipline × before/after training) were added to the LMMs to examine possible differences between GPs and nurses. Post hoc analyses were conducted for GPs and nurses separately. Subsequently, it was investigated which variables were associated with SDM support. Characteristics relating to APs’ decision-making preferences (i.e., preferred decisional role, striving for quality and length of life), APs’ similarities with the SPA patient (i.e., age, sex, patient v. survivor, palliative v. curative, corresponding type of cancer [yes/no], chemotherapy [yes/no]), SPA HCP characteristics (i.e., sex, discipline, years of experience), and APs’ video engagement (i.e., VES subscale immersion and emotional impact, ever had such a conversation, comparability) were added to the model as fixed factors one by one and maintained if *P* < 0.20. Nonsignificant variables (*P* > 0.05) were eliminated from the model one by one for simplification. All analyses were performed in IBM SPSS Statistics 26 (ICM, Amonk, NY).

## Results

### Participant and Video Details

APs (*N* = 132) were recruited through PanelCom (*n* = 106, response rate 23.6%), *n* = 19 through Kanker.nl (response rate 7.6%), and *n* = 7 by snowballing. Reasons for nonresponse were unknown. One AP was excluded from analyses as the VES screener item was ≤2, making the final sample size *N* = 131. Most APs were aged 60 to 69 y (47.3%), female (60.3%), survivors (58.0%), and more than half had received chemotherapy (61.1%). Within the group of participants who were currently having cancer, most were in the palliative phase (80.0%). See Table 1 for more participant details. As one respondent did not meet the screener criterion of the VES and was excluded from further analyses, video recordings of SPAs ultimately were assessed by three (*n* = 1), four (*n* = 27) or five (*n* = 4) APs. Ninety-three percent (*n* = 122) of the respondents had watched their assigned SPA in full. See details in Appendix C.

**Table 1:** Descriptives

Analogue patient characteristics (*N* = 131)^a^	
Cancer patient, *n* (%), v. survivor	55 (42.0)
I can still be cured	6 (10.9)
I (probably) cannot be cured	44 (80.0)
I don’t know	5 (9.1)
Years since diagnosis, $\bar{x}$ (*s*)	9.17 (6.11)
Type of cancer, *n* (%)	
Breast cancer	33 (25.2)
Colon cancer	21 (16.0)
Prostate cancer	13 (9.9)
Lymphoma	12 (9.2)
Leukemia	11 (8.4)
Esophageal cancer	6 (4.6)
Multiple myeloma	6 (4.6)
Lung cancer	5 (3.8)
Stomach cancer	2 (1.5)
Skin cancer	2 (1.5)
Other type of cancer	20 (15.3)
Type of treatment, *n* (%) (multiple answers possible)	
Surgery	93 (71.0)
Chemotherapy	80 (61.1)
Radiation	68 (51.9)
Hormonal therapy	33 (25.2)
Immunotherapy	21 (16.0)
Targeted therapy	13 (9.9)
Stem cell transplantation	6 (4.6)
Other type of treatment	4 (3.1)
No treatment	6 (4.6)
Age, y, *n* (%)	
50–59	36 (27.5)
60–69	62 (47.3)
70–79	31 (23.7)
80 or older	2 (1.5)
Sex, *n* (%) female	79 (60.3)
Educational level, *n* (%)	
Low (ISCED 0–2)	20 (15.3)
Medium (ISCED 3–4)	35 (26.7)
High (ISCED 5–8)	76 (58.0)
Preferred decisional role, *n* (%)	
Active	44 (33.6)
Shared	74 (56.5)
Passive	13 (9.8)
Tradeoff between quality and quantity of life, $\bar{x}$ (*s*)	
Striving for length of life (0–16)	6.66 (3.06)
Striving for quality of life (0–16)	12.32 (2.75)
Recruitment source, *n* (%)	
PanelCom	105 (80.2)
Kanker.nl	19 (14.5)
Snowballing	7 (5.3)
Video recording characteristics (*N* = 131)	
Ever had a similar conversation, *n* (%) yes	51 (38.9)
Comparability with own experience (1–10), $\bar{x}$ (*s*)	4.96 (2.62)
Video engagement (1–7), $\bar{x}$ (*s*)	4.81 (1.22)
Immersion	4.45 (1.42)
Emotional impact	5.18 (1.17)

ISCED, international standard classification of education; *s*, standard deviation; VES, Video Engagement Scale; $\bar{x}$ , mean.

a A total of 132 APs participated, but 1 respondent did not meet the screener criterion of the VES and was excluded from furter analyses.

### Evaluating SPAs before and after Training

APs did not significantly differ in their assessments of SPAs of HCPs before and after training on both primary outcomes. Effect sizes were small (Table 2). The intraclass correlations between APs for SDM support reflected poor agreement (*SDM support – HCP behavior*: 0.17 (before training) and −0.02 (after training); *SDM support – patient benefit*: −0.01 (before training) and 0.13 (after training)). Correlations between trained observers’ and APs’ assessments were low as well (−0.08 ≤ correlation ≤ 0.48; 0.055 ≤ *P* ≤ 0.982). The secondary outcomes did not differ significantly between SPAs before and after training either (*P* values ranging from 0.361 to 0.545 with very small negative effect sizes).

**Table 2:** Raw Means (*s*) and Linear Mixed Model Outcomes^a^

			Effect of Training (before/after)			
Outcomes (Range)	T0^b^ (*n* = 65)	T2^b^ (*n* = 66)	*b* (95% CI)	*F* (*df*)	Significance	*d* ^c^
SDM support – HCP behavior (0–5)	2.84 (1.08)	2.98 (1.14)	0.14 (−0.24, 0.52)	0.508 (1, 115.961)	0.48	0.06
SDM support – patient benefit (0–5)	2.69 (1.10)	2.72 (1.11)	0.03 (−0.35, 0.41)	0.019 (1, 115.266)	0.89	0.01
SDM support – overall (1–10)^d^	6.02 (2.18)	5.76 (2.62)	−0.26 (−1.08, 0.57)	0.381 (1, 131)	0.54	−0.05
CollaboRATE (0–100)^d^	68.44 (24.32)	65.44 (26.02)	−3.00 (−11.64, 5.64)	0.472 (1, 131)	0.49	−0.06
PSQ (0–100)	64.71 (23.98)	60.89 (26.37)	−3.87 (−12.23, 4.49)	0.840 (1, 115.517)	0.36	−0.08
Decision-making satisfaction (0–100)	63.05 (28.39)	59.09 (32.35)	−3.98 (−14.32, 6.35)	0.582 (1, 115.696)	0.45	−0.07
Conversation appreciation (0–100)	65.31 (30.24)	61.27 (33.55)	−4.12 (−14.75, 6.51)	0.590 (1, 115.659)	0.44	−0.07
Conversation helpfulness (0–100)	57.15 (29.89)	53.15 (31.73)	−3.93 (−13.91, 6.05)	0.608 (1, 115.598)	0.44	−0.07
Decision-making confidence (0–100)	67.49 (24.87)	64.88 (28.13)	−2.67 (−11.40, 6.05)	0.368 (1, 115.621)	0.54	−0.05

CI, confidence interval; HCP, health care professional; PSQ, Patient Satisfaction Questionnaire; *s*, standard deviation; SDM, shared decision making.

a These were outcomes of basic models without any covariates or interaction terms.

b T0: before training; T2: after training.

c Cohen’s *d* was calculated by $b/(\sqrt{n}*\mathrm{SE})$ .

d The variance at the HCP level was 0, and the random factor for HCP level was removed from the mixed model.

### Differences between HCP Disciplines

The size of the effect of the training (before/after) on perceived *patient benefit* as well as the *overall rating of SDM support* differed significantly between GPs and nurses (interaction term HCP discipline × before/after training: *P* = 0.034 and *P* = 0.049, respectively). Post hoc analyses within each HCP discipline separately showed that effect sizes for GPs were larger than those for nurses (Cohen’s *d* = 0.17 v. −0.06 and 0.20 v. −0.18, respectively), but none were significant. None of the effects on the other primary and secondary outcomes differed between disciplines (Table 3).

**Table 3:** Differences between GPs and Nurses

Outcome (Range)	Time × HCP Group Significance	GPs (*n* = 67)						Nurses (*n* = 64)					
		Training Effect (before/after)						Training Effect (before/after)					
		T0^a^ (*n* = 33)	T2^a^ (*n* = 34)	*b* (95% CI)	*F* (*df*)	Significance	*d* ^b^	T0^a^ (*n* = 32)	T2^a^ (*n* = 32)	*b* (95% CI)	*F* (*df*)	Significance	*d* ^b^
SDM support – HCP behavior (0–5)	0.171	2.72 (1.16)	3.12 (1.16)	0.39 (−0.16, 0.94)	2.010 (1, 59.232)	0.162	0.17	2.97 (1.01)	2.83 (1.13)	−0.13 (−0.65, 0.39)	0.253 (1, 56.677)	0.62	−0.06
SDM support – patient benefit (0–5)^c^	0.034	2.60 (1.09)	3.02 (1.08)^d^	0.42 (−0.10, 0.94)	2.584 (1, 67)	0.113	0.20	2.78 (1.13)	2.39 (1.07)^d^	−0.39 (−0.92, 0.15)	2.119 (1, 56.285)	0.151	−0.18
SDM support – overall (1–10)^c^	0.049	5.73 (2.13)	6.26 (2.50)	0.54 (−0.58, 1.65)	0.928 (1, 67)	0.34	0.12	6.31 (2.22)	5.22 (2.69)	−1.09 (−2.31, 0.12)	3.255 (1, 64)	0.076	−0.23
CollaboRATE (0–100)^c^	0.107	64.76 (26.34)	68.63 (21.58)	3.87 (−7.68, 15.42)	0.447 (1, 67)	0.51	0.08	72.23 (21.80)	62.04 (30.02)	−10.19 (−23.08, 2.71)	2.490 (1, 64)	0.119	−0.20
PSQ (0–100)	0.061	65.30 (22.86)	69.09 (21.94)^d^	3.79 (−6.93, 14.51)	0.500 (1, 59.115)	0.48	0.09	64.12 (25.45)	52.18 (28.17)^d^	−11.95 (−24.78, 0.89)	3.477 (1, 56.254)	0.067	−0.23
Decision-making satisfaction (0–100)^c^	0.22	64.00 (26.92)	66.29 (27.88)	2.29 (−10.88, 15.46)	0.121 (1, 67)	0.73	0.04	62.06 (30.22)	51.44 (35.34)	−10.65 (−26.73, 5.43)	1.760 (1, 56.144)	0.190	−0.17
Conversation appreciation (0–100)^c^	0.078	67.55 (27.30)	72.62 (28.92)^d^	5.07 (−8.44, 18.59)	0.561 (1, 67)	0.46	0.09	63.00 (33.28)	49.22 (34.33)^d^	−13.79 (−30.20, 2.61)	2.837 (1, 56.287)	0.098	−0.21
Conversation helpfulness (0–100)	0.066	59.88 (26.93)	64.82 (28.11)^d^	4.98 (−8.05, 18.02)	0.584 (1, 59.299)	0.45	0.09	54.34 (32.87)	40.75 (31.01)^d^	−13.42 (−28.50, 1.67)	3.172 (1, 56.420)	0.080	−0.22
Decision-making confidence (0–100)	0.23	70.06 (22.52)	72.56 (22.82)^d^	2.50 (−8.34, 13.33)	0.213 (1, 59.473)	0.65	0.06	64.84 (27.19)	56.72 (31.18)^d^	−8.15 (−22.06, 5.75)	1.379 (1, 56.233)	0.25	−0.15

CI, confidence interval; GP, general practitioner; HCP, health care professional; PSQ, Patient Satisfaction Questionnaire; SDM, shared decision making.

a T0: before training; T2: after training.

b Cohen’s *d* was calculated by $b/(\sqrt{n}*\mathrm{SE})$ .

c The variance at the HCP level was 0, and the random factor for HCP level was removed from the mixed model; for all variables except for SDM support – overall and CollaboRATE, the variance was only 0 for the GP group and removed from that model.

d Significant differences between GPs and nurses (*P* < 0.05).

### Variables Associated with SDM Support

Table 4 presents the variables associated with *SDM support – HCP behavior* and *patient benefit*, respectively. For both outcomes, only comparability with previous personal experiences with conversations with a GP or nurse about cancer treatment decisions (if any) was significantly associated, *F*(1, 51) = 18.34, *P* < 0.001, *d* = 0.60; *F*(1, 51) = 15.73, *P* < 0.001, *d* = 0.56. This implies that the more comparable the SPA was with the AP’s own experiences with conversations with GPs or hospital nurses about the cancer treatment decision, the higher the AP’s assessment of the SPA.

**Table 4:** Final Model SDM Support

	HCP Behavior		Patient Benefit	
	*b* (95% CI)	Significance	*b* (95% CI)	Significance
Intercept	1.75 (1.08, 2.42)	0.000	1.57 (0.90, 2.25)	0.000
Training condition				
Before (ref)				
After	−0.11 (−0.67, 0.44)	0.69	−0.22 (−0.79, 0.34)	0.43
Comparability	0.21 (0.11, 0.32)	0.000	0.23 (0.12, 0.34)	0.000

Intercept = average SDM support score of a (hypothetical) subject scoring 0 for each variable in the model. CI, confidence interval; HCP, health care professional; SDM, shared decision making.

## Discussion

This innovative, experimental study demonstrated that cancer patients and survivors (i.e., APs) did not differentiate trained from untrained HCPs when evaluating SDM support. Although trained observers established a medium effect of the training on SDM support behavior of HCPs,^21^ this effect was apparently not sufficiently large to be perceived by APs or it was not meaningful enough to them. In line, we found low correlations between assessments of trained observers and APs. This corroborates previous research on SDM as rated by observers and patients.^20,22,23,41–44^ APs may assess and prioritize differently than observers do, underscoring the necessity of evaluating outcomes on the patient level in clinical practice.^45,46^ Hence, the clinical relevance of the training program still needs to be established.^46,47^ Possibly, training programs and evaluation outcomes of this nature should be better adapted to patients’ wishes and needs by, for example, developing training programs and evaluation criteria in co-creation with patients.

APs were not more satisfied with the communication of trained HCPs. Moreover, satisfaction scores in general were lower compared with previous studies in a clinical setting (e.g., in internal medicine outpatient consultations)^38^ and in SDM conversations with oncologists.^48^ Possibly, APs are more critical of shown communication, as they are not evaluating their own doctor. Besides, not all patients may appreciate involvement of GPs or hospital nurses in decision making about cancer care, be it before or after a training. Previous research has indicated various patient experiences regarding involvement of GPs and nurses: patients reported experiencing decision support by GPs as comforting^49^ and valuable for SDM,^24^ but they also prefer or expect specialist-led care.^50^ Moreover, exposing patients to SDM means exposing them to uncertainty and responsibility, which may not be beneficial to all patients.^25,51^ In the current study, a few APs indicated in their open answers confusion about the responsibilities of medical specialists and HCPs such as GPs or nurses. Some felt that topics discussed were the responsibility of the medical specialist, as they initially discuss and eventually make the treatment decision with the patient. In addition, some APs worried that the conversation might have raised more questions and uncertainty. Patients’ varying experiences regarding SDM support were corroborated by the poor agreement between APs’ assessments in this study, which was much lower compared with the agreement between trained observers in the previous study evaluating the training’s effectiveness (Triple-S: 0.67).^21^ This stresses the importance of tailoring the amount and content of SDM conversations to patients’ needs and wishes. Future research should look further into different patients’ experiences and needs regarding SDM support.

Although both GPs and nurses are important to support SDM in the interprofessional SDM process, they have different responsibilities and expertise in the health care system.^7,52^ APs assessed GPs’ SDM support behavior typically higher, which corresponds with findings of the prior training evaluation by observers. Possibly, APs may more clearly perceive SDM support by GPs or, in some way, value it higher. Another possible explanation may be that the SPA cases may have been less applicable to the individual nurse’s situation, as nurses’ responsibilities differ largely between functions and hospitals. For example, some nurses have more decisional responsibility or more knowledge about certain cancer types than others, because of different organizational structures. This may have caused that the conversations for nurses were less appropriate and comfortable, which APs may have sensed in some way.

In addition to the fact that APs differ largely in their assessments and appreciation of SDM support, other reasons for their lack of distinction between trained and untrained HCPs may be of a more methodological nature. The current absence of measures to asses APs’ evaluation of consultation recordings made us use self-developed, nonvalidated measures as primary outcome (see Appendix B) and validated measures not designed for use in a study among APs. These measures could have been too insensitive to pick up an effect. An additional limitation worth mentioning may be selection bias, as only cancer patients and survivors interested in (patient-provider communication) research participated. For example, most had a high level of education, and compared with a 2015 review,^30^ the share of patients who wanted an active (34% v. 28%) or shared (57% v. 44%) decisional role was somewhat higher in our sample. Related, a bias may be present in the randomly selected sample of HCPs who were part of the current study. When compared with the total sample of HCPs in the prior training evaluation study, this smaller sample had similar overall effect sizes on SDM support scores as rated by trained observers, but effect sizes were relatively low for nurses and high for GPs when compared with the full sample. Therefore, conclusions on differences between GPs and nurses should be interpreted cautiously. In addition, the a priori SDM support experience of HCPs was unknown. Lastly, it remains uncertain how engaged APs were while watching the SPAs.

Next to the aforementioned methodological limitations, APs may have reported their satisfaction with general communication skills of HCPs rather than SDM support behavior, as was reported in previous research.^53^ The training program did not pay extensive attention to such general communication skills. Besides, although we used standardized patients in the SPAs, these conversations varied considerably in content other than SDM support. Also, responses between the different actors varied. Other issues may be related to APs’ engagement and ability to put oneself in the shoes of the patient in the video, although VES scores were comparable with previous research based on video recordings.^32^ The simulated context, the content, as well as the online modality of SPAs may not have been representative of APs’ personal experiences. Similarly, the sometimes suboptimal quality of the recorded SPAs may have affected immersion. The implications of video quality on APs’ immersion and perceptions are not yet empirically tested.^54^ Related, in clinical practice, SDM support is often part of a larger SDM process with multiple conversations and HCPs.^13,55^ Since the SPA only showed 1 conversation with 1 HCP, it may have been complicated for APs to consider whether this SDM support benefited the patient in their specific context and in the long run.

This innovative, experimental study design, having APs assess SPAs used for the evaluation of a training program, should be further developed and refined. A strength is the possibility to standardize the context and the patient characteristics in an SPA, ensuring all variation is attributable to the HCP. Moreover, the AP design increased the reliability of ratings, as multiple APs could assess the same HCP, and allowed for reuse of collected data and a comparison of APs’ assessments with observers’ assessments. A possible disadvantage is that there may be large differences between APs’ hypothetical and patients’ actual experiences regarding SDM support. APs may also have (unconsciously) included previous experience when assessing the conversations, which is supported by the fact that the one variable associated with their evaluation of SDM support was comparability with previous experiences. Patients who did not have a comparable experience were more critical, which may imply they used their own experience as the standard. Interesting research topics could be to explore whether APs can better differentiate trained HCPs if a larger training effect was established by observers, making the topic of interest more clearly visible, and, if possible in terms of the general data protection regulation, recordings of clinical consultations with real patients could be used to increase representativeness and immersion. Also, due to the length of the SPA recordings, APs in the current study were asked to assess only 1 recording, limiting within-AP comparisons. This is an important issue in view of the low correlation between assessments of APs who watched a similar video. Future studies could explore if patients are able to discriminate untrained from trained HCPs when watching multiple recordings.

## Conclusion

Training GPs and hospital nurses did not affect cancer patients’ and survivors’ perceived SDM support when assessing video-recorded simulated consultations. This conclusion should be drawn with caution due to the innovative study design with associated limitations, among which using a non-validated primary outcome. Since this was one of the first studies aiming at gaining insight in patient perceptions regarding SDM support in an experimental study design, future research on this topic is needed as well as further development of the study design.

### Implications

As patients did not pick up any training effect, the clinical relevance of training in SDM support for GPs and nurses still remains to be established. The large variation in cancer patients’ and survivors’ assessments of SDM support suggests patients differ in their perception of SDM support. Hence, HCPs should tailor their (offering of) SDM support to patients’ needs and wishes. It is important to create a better understanding of the reasons underlying this study’s findings. Future research should develop validated measures and explore experiences of patients with SDM support by means of other research methodologies. Qualitative studies would provide more insight into patients’ perceptions of SDM support from GPs and nurses, for example, by having APs thinking aloud while watching an SPA. Such qualitative studies might also show how the training program could be improved to meet the needs of patients. In addition, studies with video vignettes (i.e., videos that are systematically manipulated for characteristics of interest and respondents’ judgments being elicited by questionnaires)^56,57^ could systematically explore whether and what characteristics of SDM support patients prefer.

## Supplemental Material

sj-docx-1-mdm-10.1177_0272989X231203693 – Supplemental material for Through the Eyes of Patients: The Effect of Training General Practitioners and Nurses on Perceived Shared Decision-Making Support Click here for additional data file. Supplemental material, sj-docx-1-mdm-10.1177_0272989X231203693 for Through the Eyes of Patients: The Effect of Training General Practitioners and Nurses on Perceived Shared Decision-Making Support by Danique W. Bos – van den Hoek, Ellen M. A. Smets, Rania Ali, Dorien Tange, Hanneke W. M. van Laarhoven and Inge Henselmans in Medical Decision Making
