# Supplementary material for: Through the Eyes of Patients: The Effect of Training General Practitioners and Nurses on Perceived Shared Decision-Making Support
Source: Med Decis Making. 2023 Oct 24;44(1):76–88. doi: 10.1177/0272989X231203693 (PMC10714703; doi:10.1177/0272989X231203693)
Supplement: sj-docx-1-mdm-10.1177_0272989X231203693 – Supplemental material for Through the Eyes of Patients: The Effect of Training General Practitioners and Nurses on Perceived Shared Decision-Making Support [file sj-docx-1-mdm-10.1177_0272989X231203693.docx]

**Appendix A. Standardised patient assessment (SPA) cases**

**Cases that were provided to the participants**

**Case 1. Mr. van Beek**

*GPs*

Mr. van Beek (58 years old) will visit you for a (digital) consultation. He has a gastric carcinoma that has metastasised to his shoulder. Mr. van Beek has been visiting your practice for about 15 years now. He consulted you about having symptoms relating to hypertension about five years ago. You remember that you talked about his lifestyle at the time: He works a lot and likes to drink a few glasses of wine at the weekend. Back then, you advised him to take up a sport and you prescribed Metoprolol. A few weeks ago, mister van Beek came by a few times for research. When the complaints did not disappear, you referred him to the hospital. You have received a letter from the hospital’s medical oncologist. Subsequently, you invited mr. van Beek to discuss the diagnosis and treatment. Have the conversation as you normally would. The conversation has the duration of a double consultation (20 minutes). During this consultation, no physical examination will be performed.

*Nurses*

Mr. van Beek (58 years old) was diagnosed with colon carcinoma that had metastasised to his peritoneum about nine months ago. At the time, he started first-line chemotherapy (oxaliplatin, capecitabine and bevacizumab). Mr. van Beek just had a consultation with the medical oncologist regarding the results of the latest scan that was performed. Unfortunately, the results do not look good: The metastases are growing in number and size. The consultation between the medical oncologist and mr. van Beek resulted in the decision to start a new treatment. In many hospitals, if a patient receives bad news and/or a decision is made to start a new treatment, a nurse will have a conversation with the patient to support them emotionally. The nurse can take time to discuss the conversation between the patient and the oncologist. Right now, mr. van Beek visits you to discuss the conversation with the oncologist. In preparation, you have read the information below in the medical file of mr. van Beek. You don’t know him very well. Decide for yourself what you would like to discuss. Have the conversation as you normally would. The conversation lasts a maximum of 30 minutes. In this conversation, no physical examination will be performed.

**Case 2. Mr. van Es**

*GPs*

Mr. van Es (63 years old) will visit you for a (digital) consultation. He has a PA-proven oesophageal carcinoma that has metastasised to his lungs. Mr. van Es has been a patient in your practice for quite some time, yet he does not come in for consultation often. In 2013, mr. van Es’ wife passed away from stomach cancer. You remember this period being particularly difficult for him. Mr. van Es did visit you before the referral. You immediately referred him to the hospital. Subsequently, he was examined. You have received a letter from the medical oncologist and you invited mr. van Es to discuss his diagnosis and treatment. Have the conversation as you normally would. The conversation has the duration of a double consultation (20 minutes). No physical examination will be performed.

*Nurses*

Mr. van Es (63 years old) was diagnosed with colon carcinoma that had metastasised to his liver about a year ago. At the time, he started first-line chemotherapy (oxaliplatin, capecitabine and bevacizumab). Mr. van Es just had a consultation with the oncologist regarding the results of the latest scan. It doesn’t look good, the metastases are growing in number and size. The consultation between the medical oncologist and mr. van Beek resulted in the decision to start a new treatment. In many hospitals, if a patient receives bad news and/or a decision is made to start a new treatment, a nurse will have a conversation with the patient to support them emotionally. The nurse can take time to discuss the conversation between the patient and the oncologist. Right now, mr. van Beek visits you to discuss the conversation with the oncologist. In preparation, you have read the information below in the medical file of mr. van Beek. You don’t know him very well. Decide for yourself what you would like to discuss. Have the conversation as you normally would. The conversation lasts a maximum of 30 minutes. In this conversation, no physical examination will be performed.

**Treatment possibilities for metastasised stomach and oesophagus cancer**

For patients with metastasised stomach and oesophagus cancer, the chemotherapy suggested by the oncologist will be CapOx. On average, patients without this chemotherapy live 4-5 months after diagnosis and with chemotherapy 11 months. The potential benefit is therefore 6 months on average. Side effects of this chemotherapy are blood count changes, nausea or vomiting, tiredness, diarrhoea, tingling or numbness in the fingers/feet, hand-foot syndrome and muscle cramps. An alternative treatment option is to focus treatment on relieving symptoms only (best supportive care), for example pain killers or short-term radiation. Advantages are achieving optimal quality of life, not having to visit the hospital often and not taking medicines that may make you feel sick. Disadvantages are that there is no substantial inhibition of the disease and limited chances of life extension.

**Treatment possibilities for metastasised colon cancer**

For patients with metastasised colorectal cancer, the second-line chemotherapy suggested by the oncologist was a combination of irinotecan, 5-FU, leucovorin and cetuximab. This treatment can be given to patients 8-12 times (i.e. four to five months). After this cycle, the treatment can be continued as maintenance therapy with 5-FU/folonic acid and cetuximab. The potential benefit from the chemotherapy is in the order of months but the range is large. Most common side effects of this chemotherapy are cholinergic syndrome during or shortly after administration (abdominal cramps, heavy sweating, watery eyes, salivation, visual disturbances, bradycardia), diarrhoea, general malaise, nausea and vomiting, hair loss, leukopenia, liver function disorder, stomatitis (irritated mucosa in the mouth and throat), abnormal pigmentation, skin abnormalities, hypomagnesaemia, nail abnormalities/cuticle inflammation, headache, fever, interstitial pneumonitis (rare) and fall in blood pressure. An alternative treatment option is to focus treatment on relieving symptoms only (best supportive care), for example pain killers or short-term radiation. Advantages are achieving optimal quality of life, not having to visit the hospital often and not taking medicines that may make you feel sick. Disadvantages are that there is no substantial inhibition of the disease and limited chances of life extension.

**Appendix B. Questionnaire**

*Development*

The items were developed based on SDM (support) literature and models.

*Patient benefit*. In line with the three talk model of SDM [6], three ingredients of SDM important for making high-quality treatment decisions were used to assess to what extent the AP feels he or she would benefit from the shown SDM support. High-quality decisions require patients to be 1) conscious about the presence of a choice (a result of team talk), 2) informed about the pros and cons of treatment (a result of option talk) and 3) be able to make a decision based on their preferences (a result of decision talk). Items 1a-b refer to choice awareness and making a conscious decision, 2a-b to making an informed decision and 3a-c to making a decision that matches patient preferences.

*HCP behaviour*. The second set of six items focused on the perceived behaviour of nurses and GPs, based on the three strategies they adopt to support SDM about cancer treatment decisions [16, 17]: checking the quality of a decision (1 item), complementing SDM (4 items) and enabling SDM (1) items. Item 4 refers to checking the quality of decision, items 5a-c to complementing SDM and 6 to enabling SDM. Item 5a refers to the HCP contributing to making a conscious decision, 5b to the HCP contributing to making an informed decision and 5c-I and 5c-II to the HCP contributing to making a decision that matches the patient’s preferences.

The items were discussed with the research team on multiple occasions. The questionnaire was pilot tested with four participants. This resulted in slightly adjusting the questioning on some possibly sensitive topics such as if they could still be cured, adding an open answer field where participants were invited to share comments about the study, the questionnaire or the video, and offering to get in touch for support if participants wanted to.

*The following statements ask for your opinion about the conversation between the healthcare professional (HCP) and patient you just saw. You are asked to engage with the patient in the video. Please circle the number that reflects to what extent each statement applies according to you. Your impression is most important.*

|  | *Totally disagree* | *Mostly disagree* | *Disagree* | *Agree* | *Mostly agree* | *Totally agree* |
| --- | --- | --- | --- | --- | --- | --- |
| *Patient benefit* | | | | | | |
| 1a. If you were the patient, after this conversation you would know better that you can choose from several treatment options. | 0 | 1 | 2 | 3 | 4 | 5 |
| 1b. If you were the patient, after this conversation you would know better that the treatment decision depends on what you want. | 0 | 1 | 2 | 3 | 4 | 5 |
| 2a. If you were the patient, after this conversation you would know more about the treatment options you can choose from. | 0 | 1 | 2 | 3 | 4 | 5 |
| 2b. If you were the patient, after this conversation you would know more about the pros and cons of those treatment options. | 0 | 1 | 2 | 3 | 4 | 5 |
| 3a. If you were the patient, after this conversation it would be clearer to you what you consider important in the coming period. | 0 | 1 | 2 | 3 | 4 | 5 |
| 3b. If you were the patient, after this conversation it would be clearer to you what you think of the pros and cons of the treatment(s). | 0 | 1 | 2 | 3 | 4 | 5 |
| 3c. If you were the patient, after this conversation it would be clearer to you which treatment(s) suits you best. | 0 | 1 | 2 | 3 | 4 | 5 |
| *HCP behaviour* | | | | | | |
| 4. In this conversation, the HCP asked questions about what the patient knows about the treatment choice he should make. | 0 | 1 | 2 | 3 | 4 | 5 |
| 5a. In this conversation, the HCP helped to make it clear that there is a choice between several treatment options. | 0 | 1 | 2 | 3 | 4 | 5 |
| 5b. In this conversation, the HCP helped to understand the information about the treatment options. | 0 | 1 | 2 | 3 | 4 | 5 |
| 5c-I. In this conversation, the HCP helped to find out what is important for the patient in the coming period. | 0 | 1 | 2 | 3 | 4 | 5 |
| 5c-II. In this conversation, the HCP helped to find out what the patient thinks about the pros and cons of the treatment(s). | 0 | 1 | 2 | 3 | 4 | 5 |
| 6. In this conversation, the HCP helped the patient with what needs to be done after the conversation in order to make a good treatment decision. | 0 | 1 | 2 | 3 | 4 | 5 |

| This tool is protected by copyright but is available to use, provided you: a) cite the reference in any questionnaires or publications; b) do not charge for or profit from them; and c) do not alter them. Please note that this questionnaire is not developed for use in English research (no forward-backward translation applied). Please do contact the authors if you want to use the questionnaire. Contact: [i.henselmans@amsterdamumc.nl](mailto:i.henselmans@amsterdamumc.nl) |
| --- |

| **# Video** | **# HCP** | **Type HCP** | **Time** | **# APs** | **% watched** |
| --- | --- | --- | --- | --- | --- |
| 1 | 1 | GP | T0 | 4 | 3 100%; 1 50% |
| 2 |  |  | T2 | 4 | 3 100%; 1 missing |
| 3 | 2 | GP | T0 | 4 | 4 100% |
| 4 |  |  | T2 | 4 | 4 100% |
| 5 | 3 | GP | T2 | 4 | 4 100% |
| 6 |  |  | T0 | 5 | 4 100%; 1 missing |
| 7 | 4 | GP | T2 | 4 | 4 100%; 1 25% |
| 8 |  |  | T0 | 4 | 3 100%; 1 50% |
| 9 | 5 | GP | T2 | 5 | 5 100% |
| 10 |  |  | T0 | 4 | 2 100%; 1 75%; 1 missing |
| 11 | 6 | GP | T0 | 4 | 3 100%; 1 missing |
| 12 |  |  | T2 | 5 | 5 100% |
| 13 | 7 | GP | T2 | 4 | 4 100% |
| 14 |  |  | T0 | 4 | 3 100%; 1 75%^a^ |
| 15 | 8 | GP | T0 | 4 | 4 100% |
| 16 |  |  | T2 | 4 | 4 100% |
| 17 | 9 | Nurse | T0 | 4 | 4 100% |
| 18 |  |  | T2 | 4 | 3 100%; 1 75% |
| 19 | 10 | Nurse | T2 | 4 | 4 100% |
| 20 |  |  | T0 | 4 | 4 100% |
| 21 | 11 | Nurse | T0 | 3^a^ | 3 100% |
| 22 |  |  | T2 | 4 | 4 100% |
| 23 | 12 | Nurse | T2 | 4 | 4 100% |
| 24 |  |  | T0 | 4 | 4 100% |
| 25 | 13 | Nurse | T2 | 4 | 4 100% |
| 26 |  |  | T0 | 4 | 4 100% |
| 27 | 14 | Nurse | T0 | 4 | 4 100% |
| 28 |  |  | T2 | 4 | 3 100%; 1 25% |
| 29 | 15 | Nurse | T2 | 4 | 4 100% |
| 30 |  |  | T0 | 4 | 4 100% |
| 31 | 16 | Nurse | T0 | 5 | 5 100% |
| 32 |  |  | T2 | 4 | 4 100% |

**Appendix C. Table 1. Video analytics**

*Note: Missing could mean several things, e.g. the AP did not watch or the AP did not turned on cookies. Similarly, for any other data points indicating that the video was not watched 100%, this could be due to internet connection issues*

*^a^ One AP was excluded from analyses as the VES screener item ≤ 2*

*Abbreviations: AP = analogue patient, GP = general practitioner, HCP = healhcare professional, T0 = before training, T2 = after training*
